# Emergency hernia repair in the elderly: multivariate analysis of morbidity and mortality from an Italian registry

**DOI:** 10.1007/s10029-020-02269-5

**Published:** 2020-07-31

**Authors:** M. Ceresoli, F. Carissimi, A. Nigro, P. Fransvea, L. Lepre, M. Braga, G. Costa, List of Elderly Risk Assessment and Surgical Outcome (ERASO) Collaborative Study Group endorsed by SICUT, ACOI, SICG, SICE, and Italian Chapter of WSES, F. Agresta, G. Alemanno, G. Anania, M. Antropoli, G. Argenio, J. Atzeni, N. Avenia, A. Azzinnaro, G. Baldazzi, G. Balducci, G. Barbera, G. Bellanova, C. Bergamini, L. Bersigotti, P. P. Bianchi, C. Bombardini, G. Borzellino, S. Bozzo, G. Brachini, G. M. Buonanno, T. Canini, S. Cardella, G. Carrara, D. Cassini, M. Castriconi, G. Ceccarelli, D. Celi, M. Ceresoli, M. Chiappetta, M. Chiarugi, N. Cillara, F. Cimino, L. Cobuccio, G. Cocorullo, E. Colangelo, G. Costa, A. Crucitti, P. DallaCaneva, M. Luca, A. de Manzoni Garberini, C. De Nisco, M. De Prizio, A. De Sol, A. Dibella, T. Falcioni, N. Falco, C. Farina, E. Finotti, T. Fontana, G. Francioni, P. Fransvea, B. Frezza, G. Garbarino, G. Garulli, M. Genna, S. Giannessi, A. Gioffrè, A. Giordano, D. Gozzo, S. Grimaldi, G. Gulotta, V. Iacopini, T. Iarussi, G. Laracca, E. Laterza, A. Leonardi, L. Lepre, L. Lorenzon, G. Luridiana, A. Malagnino, G. Mar, P. Marini, R. Marzaioli, G. Massa, V. Mecarelli, P. Mercantini, A. Mingoli, G. Nigri, S. Occhionorelli, N. Paderno, G. M. Palini, D. Paradies, M. Paroli, F. Perrone, N. Petrucciani, L. Petruzzelli, A. Pezzolla, D. Piazza, V. Piazza, M. Piccoli, A. Pisanu, M. Podda, G. Poillucci, R. Porfidia, G. Rossi, P. Ruscelli, A. Spagnoli, R. Sulis, D. Tartaglia, C. Tranà, A. Travaglino, P. Tomaiuolo, A. Valeri, G. Vasquez, M. Zago, E. Zanoni

**Affiliations:** 1 General and Emergency Surgery Department, School of Medicine and Surgery, University of Milano-Bicocca, via Pergolesi 33, 20900 Monza, Italy; 2 UOC Chirurgia D’Urgenza E del Trauma, Fondazione Policlinico Universitario A. Gemelli IRCCS, Università Cattolica del Sacro Cuore, Roma, Italy; 3 UOC Chirurgia Generale, Ospedale Santo Spirito in Sassia, ASL Roma 1, Roma, Italy; 4 Surgical and Medical Department of Translational Medicine, Sant’Andrea Teaching Hospital, Sapienza University of Roma, Roma, Italy

**Keywords:** Groin hernia, Incarcerated hernia, Elderly, Postoperative complications, Emergency surgery, Charlson’s comorbidity index

## Abstract

**Purpose:**

The incidence of inguinal hernia is higher in elderly because of aging-related diseases like prostatism, bronchitis, collagen laxity. A conservative management is common in elderly to reduce surgery-related risks, however watchful waiting can expose to obstruction and strangulation. The aim of the present study was to assess the impact of emergency surgery in a large series of elderly with complicated groin hernia and to identify the independent risk factors for postoperative morbidity and mortality. The predictive performance of prognostic risk scores has been also assessed.

**Methods:**

This is a prospective observational study carried out between January 2017 and June 2018 in elderly patients who underwent emergency surgery for complicated hernia in 38 Italian hospitals. Pre-operative, surgical and postoperative data were recorded for each patient. ASA score, Charlson’s comorbidity index, P-POSSUM and CR-POSSUM were assessed.

**Results:**

259 patients were recruited, mean age was 80 years. A direct repair without mesh was performed in 62 (23.9%) patients. Explorative laparotomy was performed in 56 (21.6%) patients and bowel resection was necessary in 44 (17%). Mortality occurred in seven (2.8%) patients. Fifty-five (21.2%) patients developed complications, 12 of whom had a major one. At univariate and multivariate analyses, Charlson’s comorbidity index ≥ 6, altered mental status, and need for laparotomy were associated with major complications and mortality

**Conclusion:**

Emergency surgery for complicated hernia is burdened by high morbidity and mortality in elderly patients. Preoperative comorbidity played a pivotal role in predicting complications and mortality and therefore Charlson’s comorbidity index could be adopted to select patients for elective operation

**Electronic supplementary material:**

The online version of this article (10.1007/s10029-020-02269-5) contains supplementary material, which is available to authorized users.

## Introduction

Inguinal and femoral hernias are very common clinical situations worldwide with estimated prevalence of 27–43% in men and 3–6% in women [1]. Despite groin hernia is widespread in all age groups of population, its incidence is higher in elderly [2]. Conditions frequently associated to advanced age, such as constipation, prostatism, frequent coughing due to respiratory diseases and weakness of the abdominal wall, play an important role in the development and evolution of abdominal wall hernias [2, 3].

Groin hernias can progress to incarceration and strangulation which constitute a common surgical emergency. The estimated risk of an inguinal hernia becoming incarcerated is 4.5% after 2 years and the complication risk is higher in femoral hernia with a 22% cumulative probability at 3 months and 45% at 21 months [4].

Regardless of age and frailty European Hernia Society Guidelines recommend surgery in case of symptomatic inguinal hernia; whereas if patients do not complain of symptoms the indication to surgical repair is debated, being a watchful approach an option [3]. Although elective surgery repair is performed safely with minimal morbidity [5, 6] conservative treatment is sometimes preferred in elderly due to comorbidities. On the other hand, the natural history of a conservatively managed groin hernia is size increasing due to continuous action of intra-abdominal pressure and progressive abdominal wall laxity [3]. This exposes patients to an increasing risk of bowel obstruction and strangulation requiring emergency surgery with consequent risk of laparotomy and bowel resection [7]. The balance between the risks of elective surgery versus the risks of a watchful approach is still a matter of debate in absence of specific recommendations for elderly.

The aim of the present study was to assess the impact of emergency surgery in a large series of elderly patients with complicated groin hernia and to identify independent risk factors for postoperative morbidity and mortality. The predictive performance of prognostic risk scores has also been assessed.

## Methods

The present study analyzed data from the Frailty and Emergency Surgery Study (FRAILESEL) database [8]; FRAILESEL is a prospective observational project that collected data in consecutive elderly patients who underwent emergency surgery in 38 Italian hospitals. The Study protocol was approved by the Ethics Committee of Sapienza University of Rome and of all participating centers and was registered on clinicaltrials.gov (ClinicalTrials.gov identifier: NCT02825082). All patients who underwent emergency surgery for incarcerated inguinal or femoral hernia between January 2017 and June 2018 were included in the present study. For each patient the following data were recorded: age, sex, BMI, comorbidities, American Society of Anesthesiologist (ASA) score, preoperative hemodynamic status, type of incarcerated hernia (inguinal or femoral), surgical technique, need for explorative laparotomy and bowel resection. For each patient, the Charlson’s comorbidity index [9, 10], the predicted morbidity and mortality risks according to the P-POSSUM and the CR-POSSUM models [11, 12] were also calculated.

All postoperative complications and reoperations that occurred during hospitalization or within 30 days after discharge were registered and graded according to the Clavien-Dindo classification [13].

Continuous variables were expressed as mean (SD) or median (IQR) as appropriate; categorical data were showed as proportion and percentages. Five different variables were selected as outcomes: explorative laparotomy, abdominal viscera resection, complications, major complications (Clavien-Dindo ≥ IIIb), and mortality. Univariate analysis was carried out with the chi square test and Mann–Whitney *U* test; variables significantly associated with the outcomes were inserted in a multivariate model with the logistic regression method; multivariate analysis was not computed in case of number of events < 10.The ASA scores and the Charlson’s comorbidity index were analyzed with the ROC curves method in order to choose a cut-off for complications, major complications and mortality. ASA score (both as categorical and with the cut-off chosen with the ROC method), the Charlson’s comorbidity index (both as continuous, categorical the cut-off chosen with the ROC method), the predicted risk of morbidity and mortality with the P-POSSUM and CR-POSSUM models and the length of stay were compared among patients with or without the selected outcomes (morbidity and mortality) with the appropriate test. Statistics were calculated with SPSS 25 IBM Corp. Released 2017. IBM SPSS Statistics for Windows, Version 25.0. Armonk, NY: IBM Corp.).

## Results

A total of 259 consecutive patients operated for complicated inguinal or femoral hernia were included in the analysis.

Table 1 reports patients’ characteristics in detail. Mean age was 80(± 8) years and 58% patients were men. Common comorbidities were hypertension (65%), chronic heart disease (28%), arrhythmia (34%), and COPD (18%) while 20% of patients were in therapy with oral anticoagulants. Patients with inguinal hernia were similar to those with femoral hernia in terms of comorbidity and pre-operative characteristics; as expected female sex was more common in femoral hernia (84% vs. 23%, *p* < 0.001).

**Table 1:** Patients characteristics

	Mean (SD)	Median (IQR)	*N*	%
Number of patients			259	
Age	79.70 (8.37)	79 (73–87)		
Age class				
65–70			41	15.1%
71–75			49	18.3%
76–80			51	19.7%
81–85			39	15.1%
86–90			51	19.7%
> 90			28	10.8%
Sex				
Female			109	42.1%
Male			150	57.9%
BMI	25.36 (5.67)	24.74 (22–27)		
Mental status impairment			14	6.3%
Hypotension (SBP < 90)			3	1.2%
Tachycardia (HR > 100)			14	5.4%
Comorbidity				
Atrial Fibrillation/arrhythmia			96	37.1%
Ischemic heart disease			8	3.1%
Chronic heart disease			73	28.2%
Arterial hypertension			168	64.9%
Peripheral artery disease			37	14.3%
Cerebrovascular disease			38	14.7%
Oral Anticoagulants			52	20.1%
COPD			48	18.5%
Metastatic Cancer			9	3.5%
Cancer without metastasis			23	8.9%
Leukemia/lymphoma			7	2.7%
Hepatic disease			10	3.9%
Kidney disease			22	8.5%
Diabetes			32	16.2%
Peptic ulcer			6	2.3%
Connective tissue disease			10	3.9%
Steroids/immunosuppressive			14	5.4%
Emiplegia			10	3.9%
Demenza			27	10.4%
ASA score				
1			12	4.7%
2			88	34.8%
3			132	52.2%
4			20	7.9%
5			1	0.4%
Charlson comorbidity index	4.97 (2.28)	4 (3–6)		
Charlson comorbidity index				
< 6			176	68%
≥ 6			83	32%
Charlson comorbidity index				
0–1			0	0%
2–3			71	27%
4–5			105	40.5%
6–7			46	17.8%
8–9			28	10.8%
10–11			5	1.9%
12–13			2	0.8%
14–15			1	0.4%
16–17			1	0.4%
Predicted mortality risk (PPOSSUM)	7.81 (12.46)	3.60 (1.5–8.1)		
Predicted morbidity risk (PPOSSUM)	50.68 (24.11)	49 (29–71)		
Predicted mortality risk (CR-POSSUM)	6.88 (7.34)	5.10 (1.9–9)		

Table 2 shows surgical data and outcomes. One hundred and eighty (69.5%) patients were operated for inguinal hernia and 79 (30.5%) for femoral hernia. Laparoscopic surgery was carried out in 10 (12.66%) patients. A mesh repair was performed in 91% of patients with inguinal hernia and in 41% with femoral hernia. At univariate analysis, factors related to the mesh placement were increasing BMI (as a continuous variable) (OR = 1.147; CI 95% = 1.040–1.264); male gender (OR = 5.501; CI 95% = 2.922–10.35); femoral hernia (OR = 0.062; CI 95% = 0.031–0.124), need for explorative laparotomy (OR = 0.379; CI 95% = 0.200–0.718) and bowel resection (OR = 0.230; CI 95% = 0.119–0.446). At multivariate analysis only femoral hernia maintained an independent association with mesh (OR = 0.64; CI 95% = 0.021–0.199).

**Table 2:** Surgery data and outcomes

	Mean (SD)	Median (IQR)	*n* (%)	%
Time to surgery (days)	0.58 (1.49)	0 (0–1)		
Kind of hernia				
Inguinal			180	69.11%
Femoral			79	30.12%
Inguinal hernia				
Direct repair			15	8.33%
Mesh			165	91.67%
Femoral hernia				
Direct repair			47	59.49%
Mesh			32	40.51%
Laparoscopic repair			10	12.66%
Explorative laparotomy/laparoscopy			56	21.62%
Intestinal resection				
No			215	83.01%
Colon			2	0.77%
Ileum			41	15.83%
Ileum-cecum			1	0.39%
Length of stay	5.17 (4.02)	4.00 (2.00–7.00)		
Reintervention			3	1.16%
Major complications			12	4.63%
Complications			55	21.24%
Perforation			2	0.77%
Occlusion			5	1.93%
Pneumonia			8	3.09%
Acute renal failure			4	1.54%
Bleeding			5	1.93%
Stroke			2	0.77%
Acute myocardial infarction/heart failure			3	1.16%
Arrhythmia			5	1.93%
SSI			6	2.32%
Mortality			7	2.70%

An explorative laparotomy was necessary in 56 (21.6%) patients and 44 (17.0%) of them had a bowel resection. At multivariate analysis, significant risk factors were ASA score > 2 for laparotomy and femoral hernia for bowel resection (Table 3).

**Table 3:** Univariate and multivariate analysis of factor associated to laparotomy and resection

	laparotomy				Resection			
	OR (95% CI) univariate	*p*	OR (95% CI) multivariate	*p*	OR (95% CI) univariate	*p*	OR (95% CI) multivariate	*p*
Age	1.006 (0.971–1.042)	0.736			0.984 (0.948–1.022)	0.399		
BMI	0.974 (0.906–1.048)	0.482			0.941 (0.859–1.031)	0.193		
Male sex	0.606 (0.334–1.098)	0.097			0.784 (0.42–1.464)	0.445		
ASA ≥ 3	**3.16 (1.57–6.33)**	**0.001**	**1.876 (1.165–3.021)**	**0.01**	1.94 (0.991–3.831)	0.051		
Charlson ≥ 6	1.66 (0.901–3.065)	0.102			1.44 (0.758–2.754)	0.262		
Femoral Hernia (inguinal ref)	1.830 (0.989–3.384)	0.052			**2.187 (1.153–4.147)**	**0.015**	**2.275 (1.190–4.348)**	**0.013**
Arrhythmia	1.241 (0.678–2.227)	0.483			1.216 (0.644–2.296)	0.546		
Myocardial infarction	1.216 (0.239–6.196)	0.814			1.447 (0.283–7.395)	0.656		
Chronic heart disease	**3.882 (1.082–13.925)**	**0.026**	2.260 (0.577–8.846)	0.242	3.022 (0.819–11.153)	0.083		
Hypertension	0.858 (0.464–1.586)	0.625			0.652 (0.346–1.231)	0.186		
Cerebrovascular disease	1.359 (0.616–2.999)	0.447			0.776 (0.305–1.974)	0.594		
Oral anticoagulants	1.11 (0.538–2.297)	0.776			1.193 (0.562–2.533)	0.645		
Chronic lung diseases	1.657 (0.816–3.364)	0.159			1.568 (0.745–3.279)	0.233		
Metastatic solid tumors	1.858 (0.45–7.679)	0.385			**3.644 (1.041–14.112)**	**0.047**	**4.008 (1.029–16.24)**	**0.045**
Non-metastatic solid tumors	0.745 (0.243–2.286)	0.606			0.62 (0.177–2.1734)	0.451		
Liver disease	0.903 (0.186–4.376)	0.899			1.891 (0.471–7.592)	0.362		
Kidney disease	1.073 (0.378–3.047)	0.895			0.948 (0.306–2.938)	0.926		
Diabetes	0.440 (0.164–1.178)	0.095			0.402 (0.136–1.186)	0.089		
Steroids/immunosoppressors	1.485 (0.447–4.926)	0.516			2.538 (0.811–7.942)	0.099		
Dementia	**2.857 (1.241–6.577)**	**0.011**	2.051 (0.841–5.001)	0.114	1.961 (0.804–4.787)	0.133		
Leukemia/lymphoma	0.597 (0.07–5.064)	0.633			1.745 (0.328–9.270)	0.509		

Bold indicate depicted significative results

Post-operative outcomes are reported in Table 2. Overall morbidity was 21.2%. Major complications occurred in 12 (4.6%) patients and mortality in seven (2.8%) patients. Three patients died for sepsis, one for heart failure, acute cardiac ischemia, stroke, and hemorrhage. Mean length of stay was significantly longer in patients with complications than in uneventful (8.3 vs. 4.3, *p* < 0.001) (Table 4).

The multivariate analysis demonstrated that preoperative conditions, such as heart and lung dysfunctions and Charlson’s comorbidity index ≥ 6, were independently associated with major complications and mortality (Table 5).

**Table 4:** Prognostic score assessment and distribution among patients

	Complications				*p*-value	Major complications				*p*-value	Mortality				*p*-value
	No		Complicated			None		Complicated			Alive		Dead		
	*N*/mean	%/(SD)	*N*/mean	%/(SD)		*N*/mean	%/(SD)	*N*/mean	%/(SD)		*N*/mean	%/(SD)	*N*/mean	%/(SD)	
ASA score															
Mean	2.59	0.70	2.84	0.74	0.024	2.62	0.69	3.08	1.00	0.024	2.61	0.69	3.57	0.98	< 0.001
ASA score															
1	11	91.7%	1	8.3%	0.105	12	100.0%	0	0.0%	< 0.001	12	100.0%	0	0.0%	< 0.001
2	72	81.8%	16	18.2%		84	95.5%	4	4.5%		85	98.8%	1	1.2%	
3	102	77.3%	30	22.7%		128	97.0%	4	3.0%		126	98.4%	2	1.6%	
4	13	65.0%	7	35.0%		17	85.0%	3	15.0%		16	84.2%	3	15.8%	
5	0	0.0%	1	100.0%		0	0.0%	1	100.0%		0	0.0%	1	100.0%	
ASA score															
< 3	89	84.0%	17	16.0%	0.089	102	96.2%	4	3.8%	0.584	103	99.0%	1	1.0%	0.141
≥ 3	115	75.2%	38	24.8%		145	94.8%	8	5.2%		142	95.9%	6	4.1%	
CHARLSON															
Mean	4.78	2.05	5.76	2.76	0.004	4.91	2.11	6.67	3.98	0.008	4.92	2.11	8.86	3.85	< 0.001
Charlson comorbidity index															
< 6	146	83.0%	30	17.0%	0.016	171	97.2%	5	2.8%	0.046	169	100.0%	0	0.0%	< 0,001
≥ 6	58	69.9%	25	30.1%		76	91.6%	7	8.4%		76	91.6%	7	8.4%	
Charlson comorbidity index															
1–2-Jan	60	84.5%	11	15.5%	0.153	69	97.2%	2	2.8%	< 0.001	67	100.0%	0	0.0%	< 0.001
4–5-Apr	86	81.9%	19	18.1%		102	97.1%	3	2.9%		102	100.0%	0	0.0%	
6–7-Jun	34	73.9%	12	26.1%		43	93.5%	3	6.5%		43	93.5%	3	6.5%	
8–9-Aug	19	67.9%	9	32.1%		26	92.9%	2	7.1%		26	92.9%	2	7.1%	
10–11-Oct	3	60.0%	2	40.0%		4	80.0%	1	20.0%		4	80.0%	1	20.0%	
12–13-Dec	1	50.0%	1	50.0%		2	100.0%	0	0.0%		2	100.0%	0	0.0%	
14–15	1	100.0%	0	0.0%		1	100.0%	0	0.0%		1	100.0%	0	0.0%	
16–17	0	0.0%	1	100.0%		0	0.0%	1	100.0%		0	0.0%	1	100.0%	
LOS	4.30	3.25	8.38	4.88	< 0.001	4.93	3.63	10.00	7.54	< 0.001	5.00	3.80	11.57	7.18	< 0.001
Predicted mortality risk (PPOSSUM) (mean)											7.18	10.91	30.70	33.30	< 0.001
Predicted morbidity risk (PPOSSUM) (mean)	46.97	22.41	64.43	25.41	< 0.001	49.98	23.85	64.96	26.01	0.035					
Predicted mortality risk (CR-POSSUM) (mean)											6.63	7.00	17.07	13.74	< 0.001

**Table 5:** Univariate and multivariate analysis for complications and mortality

	Complication				Major complication		Mortality	
	OR (95% CI) univariate	*p*	OR (95% CI) multivariate	*p*	OR (95%CI) univariate	*p*	OR (95%CI) univariate	*p*
Age	1.010 (0.974–1.046)	0.594			0.959 (0.892–1.031)	0.256	1.019 (0.931–1.115)	0.69
BMI	0.968 (0.902–1.032)	0.364			0.924 (0.784–1.089)	0.347	0.842 (0.687–1.031)	0.096
hypotension	7.843 (0.678–88.20)	0.051			**53.74 (4.45–649)**	**< 0.001**	**120 (8.95–1600)**	**< 0.001**
Tachycardia	**4.217 (1.41–12.74)**	**0.006**	2.889 (0.673–12.40)	0.154	**7.909 (1.84–33.98)**	**0.001**	**23.1 (4.13–129)**	**< 0.001**
Mental impairment	**3.837 (1.277–11.529)**	**0.011**	2.502 (0.626–9.993)	0.194	**10 (2.57–38.88)**	**< 0.001**	**26.4 (5.192–134.226)**	**< 0.001**
Male Sex	0.635 (0.349–1.155)	0.135			1.018 (0.314–3.297)	0.976	1.875 (0.357–9.854)	0.451
ASA ≥ 3	1.730 (0.916–3.265)	0.089			1.407 (0.413–4.797)	0.584	4.352 (0.516–36.70)	0.141
Charlson ≥ 6	**2.09 (1.138–3.867)**	**0.016**	1.105 (0.624–1.956)	0.732	**3.150 (1.03–10.24)**	**0.046**	**–**	**< 0.001**
Crural hernia	1.404 (0.750–2.630)	0.287			1.147 (0.335–3.925)	0.827	0.889 (0.169–4.687)	0.89
Laparotomy	**4.161 (2.166–7.995)**	**< 0.001**	**6.607 (2.905–15.03)**	**< 0.001**	**5.657 (1.722–18.586)**	**0.002**	**5.2 (1.127–23.987)**	**0.02**
Prosthesis	0.795 (0.433–1.46)	0.46			1.008 (0.285–3.571)	0.99	1.034 (0.205–5.223)	0.968
Bowel resection	**3.448 (1.755–6.776)**	**< 0.001**	0.721 (0.193–3.165)	0.728	**6.833 (2.069–22.567)**	**< 0.001**	**6.264 (1.353–29.005)**	**0.008**
Arrhythmia	**2.074 (1.134–3.792)**	**0.017**	**2.813 (1.317–6.008)**	**0.008**	1.224 (.0378–3.971)	0.735	1.247 (0.273–5.698)	0.775
Ischemic heart disease	2.296 (0.531–9.922)	0.253			**8.003 (1.437–44.897)**	**0.005**	**15.933 (2.558–99.23)**	**< 0.001**
Chronic heart disease	2.588 (0.704–9.516)	0.139			**11.429 (2.531–51.597)**	**< 0.001**	**11.850 (1.898–70.605)**	**0.001**
Hypertension	1.739 (0.890–3.397)	0.103			2.767 (0.593–12.91)	0.178	3.188 (0.378–26.91)	0.261
cerebrovascular disease	1.181 (0.522–2.668)	0.689			1.172 (0.247–5.572)	0.841	2.322 (0.434–12.42)	0.312
Oral anticoagulants	1.915 (0.966–3.795)	0.06			3.04 (0.924–9.999)	0.056	**5.617 (1.216–25.95)**	**0.014**
COPD	**2.205 (1.102–4.412)**	**0.023**	**2.505 (1.024–6.126)**	**0.044**	**3.389 (1.027–11.182)**	**0.035**	3.426 (0.74–15.855)	0.095
Metastatic solid tumors	1.904 (0.461–7.87)	0.366			2.716 (0.312–23.666)	0.347	4.938 (0.53–45.972)	0.121
Non-metastatic solid tumors	1.347 (0.504–3.598)	0.551			2.152 (0.442–10.478)	0.332	4.5 (0.82–24.692)	0.059
Liver disease	0.401 (0.05–3.237)	0.376			–	0.477	–	0.606
Kidney disease	1.838 (0.71–4.758)	0.205			2.27 (0.465–11.082)	0.298	4.5 (0.82–24.692)	0.59
Diabetes	1.868 (0.895–3.898)	0.093			1.778 (0.461–6.862)	0.398	4.086 (0.879–18.98)	0.053
Immunosuppressive drugs	**3 (1.004–9.047)**	**0.042**	3.684 (0.941–14.42)	0.061	1.636 (0.196–3.659)	0.646	2.974 (0.333–26.562)	0.306
Dementia	2.022 (0.853–4.791)	0.104			**4.87 (1.361–17.421)**	**0.008**	**13.515 (2.841–64.297)**	**< 0.001**
Leukemia/lymphoma	0.611 (0.072–5.185)	0.649			3.652 (0.404–33.006)	0.218	6.639 (0.688–64.05)	0.06

### Prognostic scores

The predicted risk according to the P-POSSUM model was 50% (± 24) for morbidity and 7.81% (± 12) for mortality. The CR-POSSUM model prediction mortality was 6.88% (± 7).

With the ROC curves method were individuated two cut-off for the ASA score (cut-off three) and Charlson’s comorbidity index (cut-off six) (see supplementary materials).

**Fig. 1:**
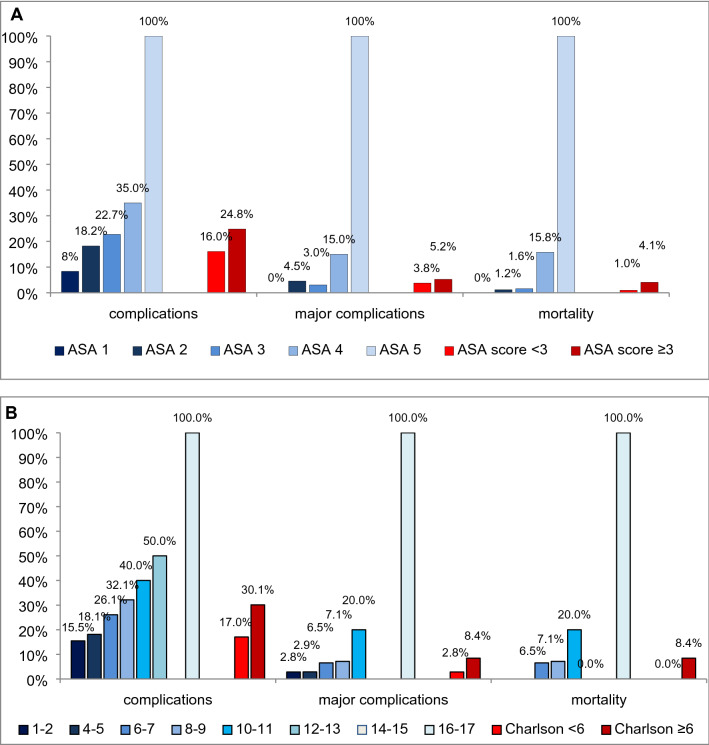
**a** complications, major complications and mortality rates among ASA score (**a**) and Charlson’s comorbidity index (**b**) classes

Major morbidity was 5.2%, in patients with ASA score ≥ 3 compared with 3.8% in patients with ASA < 3 (*p* = 0.584). In patients with Charlson’s comorbidity index ≥ 6 major morbidity was 8.4% compared with 2.8% in patients with index < 6 (*p* < 0.045). Mortality with ASA score ≥ 3 was 4.1, compared with 1% in patients with ASA < 3 (*p* = 0.141). In patients with Charlson’s comorbidity index ≥ 6 mortality was 8% compared with 0% in patients with index < 6 (*p* < 0.001). Results are shown in detail in Table 4 and Fig. 1.

## Discussion

The present study shows that emergency surgery for complicated hernia is burdened by high morbidity and mortality in elderly patients. Femoral hernia was associated with a higher risk of laparotomy and bowel resection. Heart and lung dysfunction, impaired mental status, and oral anticoagulant therapy were correlated to postoperative complications and mortality.

In current practice, elderly patients presenting with asymptomatic groin hernia are often managed conservatively to avoid the surgery-related risk of complications. A watchful waiting is recognized as an acceptable option for patients with asymptomatic or minimally symptomatic inguinal hernias [14, 15]. On the other hand, an incarcerated hernia can be sometimes difficult to identify by physical examination [16] and a delayed diagnosis could significantly increase the risk of strangulation. Incarceration and even more strangulation seldom occur, but require mandatory emergency surgery which is burdened by higher mortality and morbidity in elderly when compared to younger patients [17, 18]. In the emergency setting general anesthesia is usually preferred, whereas local or loco-regional anesthesia is the first option for elective hernia repair, especially in elderly patients with severe comorbidities [19].

In the present study the overall postoperative mortality (2.8%) was substantially higher than those reported after elective hernia repair in elderly [20]. In the subgroup of patients who had laparotomy and bowel resection mortality was 7.14%, consistent with previous series reporting a mortality increase up to 20% in case of ischemic herniated bowel resection [20–22]. Overall morbidity was 21% and major complication rate was 5%, both aligning with the existing literature on emergency surgery for complicated hernia [23], but much higher when compared to elective surgery [24].

At multivariate analysis, impaired mental status, heart and lung dysfunctions, and oral anticoagulant therapy were independently associated to major complications and mortality. Noteworthy, diabetes was not associated with morbidity or mortality in our cohort of patients. Usually, the presence of comorbidities advises physicians to prefer a watchful approach. Despite the present study cannot demonstrate the superiority of an operative approach to groin hernia due to the lack of a control population the indication to perform an elective procedure should be carefully tailored, balancing the risk of hernia incarceration and the risk of postoperative complications, in case of emergency surgery. A particular attention should be reserved to patients with oral anticoagulants that can be safely stopped in proper time in case of elective surgery, but not in emergency setting.

To predict surgical risk in patients undergoing emergency surgery for incarcerated/strangulated groin hernia some common preoperative score have been tested. ASA score and above all Charlson’s comorbidity index allowed an easy and rapid stratification of patients at high risk for morbidity and mortality. Conversely, P-POSSUM and CR-POSSUM which has been specifically validated for colorectal and major surgery, failed to predict morbidity and mortality, with a predicted risk overestimation. Therefore, ASA score and Charlson’s comorbidity index could be adopted as valid tools for risk stratification in elderly to select candidates for elective hernia repair.

In patients undergoing emergency surgery, the use of mesh to repair hernia is still an open issue because prosthesis could increase the infectious risk [25]. However, in accordance with the EHS guidelines [3], a direct repair without mesh brings a greater risk of recurrence with possible need of redo surgery. According to WSES guidelines [16] a mesh should be used in clean and clean contaminated (CDC class I and II) [13] emergency setting, while the use of mesh should be discouraged in dirty/contaminated surgery which is burdened by an infection rate up to 38% following bowel resection [26]. In the present study the only independent factor related to direct repair was femoral hernia. An high proportion of patients with femoral hernia in fact did not receive mesh positioning (59.5%), exposing them to the risk of recurrence; on the contrary a great proportion of patients operated for inguinal hernia had the positioning of a mesh, despite the presence of strangulated/incarcerated viscera and the consequent risk of infection. In our series of elderly patients factors associated with the non-positioning of mesh were explorative laparotomy and bowel resection, both indicating the presence of a contaminated surgical field. Moreover also the age could have played an important role: the lower life expectation of elderly could have mitigated the risk of recurrence linked to the direct repair.

The observational multicentre cohort design without a control population to compare is a limitation of the present study, therefore no clear recommendations could be derived from the present paper; moreover the study was not originally designed specifically for groin hernia and therefore some important information are missing like the timeframe between incarceration and presentation in hospital. However, the prospective data collection and a priori definition of criteria to identify postoperative complications might mitigate this limitation. Moreover, a multicentre study allows better generalization of results than single centre, while the large series of patients allowed excluding confounders by multiple logistic analyses.

In conclusion, emergency surgery for complicated hernia is burdened by high morbidity and mortality in elderly patients. Femoral hernia was associated with a higher risk of laparotomy and bowel resection. Since preoperative comorbidity played a pivotal role in predicting complications and mortality, Charlson’s comorbidity index should be adopted as a valid tool for evaluate and select patients for elective operation.

## Electronic supplementary material

Below is the link to the electronic supplementary material.

Supplementary file1 (DOCX 54 kb)

## Data Availability

Data are available on request to the corresponding author.
