# Supplementary material for: Emergency hernia repair in the elderly: multivariate analysis of morbidity and mortality from an Italian registry
Source: Hernia. 2020 Jul 31;26(1):165–75. doi: 10.1007/s10029-020-02269-5 (PMC8881429; doi:10.1007/s10029-020-02269-5)
Supplement: Supplementary file 1 — Supplementary file1 (DOCX 54 kb) [file 10029_2020_2269_MOESM1_ESM.docx]

ROC curves of Charlson’s comorbidity index:

Mortality:


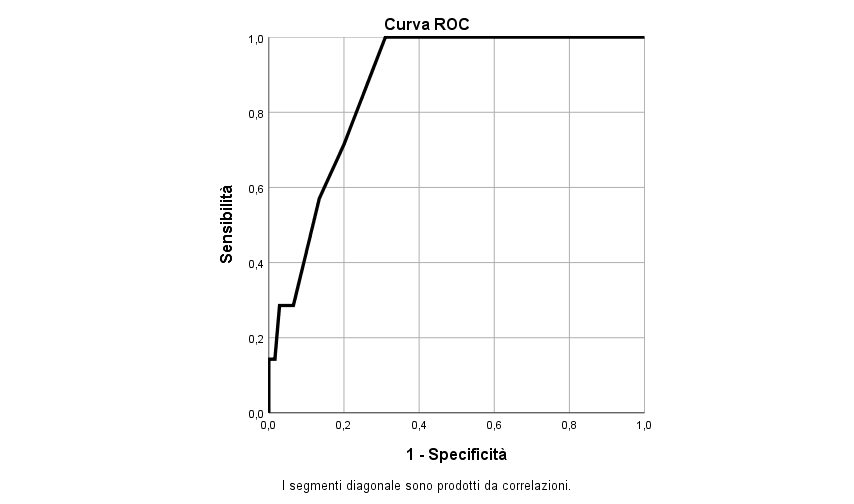


| **Area sotto la curva** | | | | |
| --- | --- | --- | --- | --- |
| Variabile/i del risultato del test: | | | | |
| Area | Errore standard^a^ | Sign. asintotica^b^ | Intervallo di confidenza asintotica 95% | |
|  |  |  | Limite inferiore | Limite superiore |
| 0,871 | 0,040 | 0,001 | 0,793 | 0,950 |
| Variabile/i del risultato del test:CHARLSON hanno almeno una correlazione tra il gruppo di stati effettivi positivi e il gruppo di stati effettivi negativi. Le statistiche possono essere distorte. | | | | |
| a. In base all'ipotesi non parametrica | | | | |
| b. Ipotesi nulla: true, area = 0,5 | | | | |

Complications


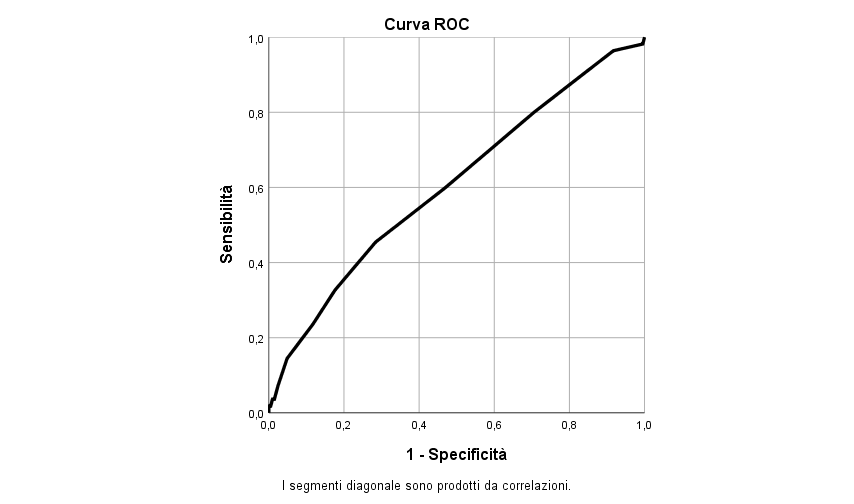


| **Area sotto la curva** | | | | |
| --- | --- | --- | --- | --- |
| Variabile/i del risultato del test: | | | | |
| Area | Errore standard^a^ | Sign. asintotica^b^ | Intervallo di confidenza asintotica 95% | |
|  |  |  | Limite inferiore | Limite superiore |
| 0,605 | 0,044 | 0,016 | 0,519 | 0,692 |
| Variabile/i del risultato del test:CHARLSON hanno almeno una correlazione tra il gruppo di stati effettivi positivi e il gruppo di stati effettivi negativi. Le statistiche possono essere distorte. | | | | |
| a. In base all'ipotesi non parametrica | | | | |
| b. Ipotesi nulla: true, area = 0,5 | | | | |

Major complications


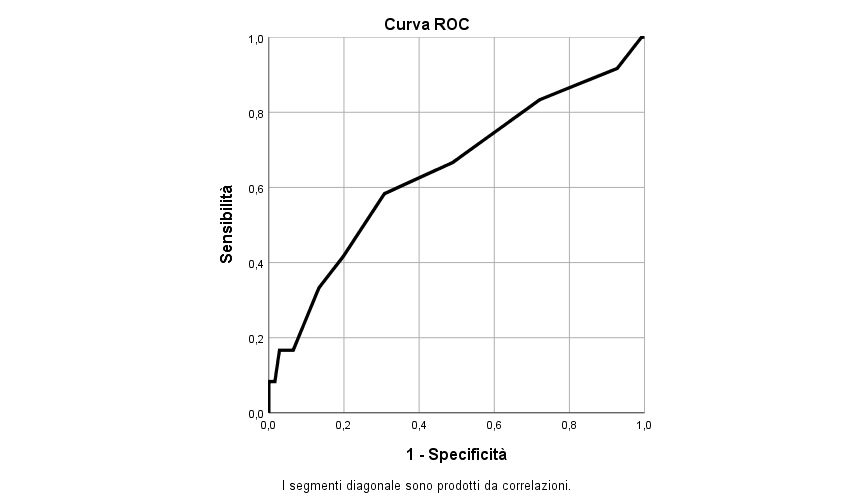


| **Area sotto la curva** | | | | |
| --- | --- | --- | --- | --- |
| Variabile/i del risultato del test: | | | | |
| Area | Errore standard^a^ | Sign. asintotica^b^ | Intervallo di confidenza asintotica 95% | |
|  |  |  | Limite inferiore | Limite superiore |
| 0,643 | 0,091 | 0,095 | 0,465 | 0,821 |
| Variabile/i del risultato del test:CHARLSON hanno almeno una correlazione tra il gruppo di stati effettivi positivi e il gruppo di stati effettivi negativi. Le statistiche possono essere distorte. | | | | |
| a. In base all'ipotesi non parametrica | | | | |
| b. Ipotesi nulla: true, area = 0,5 | | | | |
